# Neural responses to natural and enhanced speech edges in children with and without dyslexia

**DOI:** 10.3389/fnhum.2023.1200950

**Published:** 2023-09-28

**Authors:** Kanad Mandke, Sheila Flanagan, Annabel Macfarlane, Georgia Feltham, Fiona Gabrielczyk, Angela M. Wilson, Joachim Gross, Usha Goswami

**Affiliations:** ^1^Department of Psychology, Centre for Neuroscience in Education, University of Cambridge, Cambridge, United Kingdom; ^2^Institute for Biomagnetism and Biosignal Analysis, University of Münster, Münster, Germany

**Keywords:** dyslexia, magnetoencephalography, neural oscillations, speech processing, rise time, phonological deficit

## Abstract

Sensory-neural studies indicate that children with developmental dyslexia show impairments in processing acoustic speech envelope information. Prior studies suggest that this arises in part from reduced sensory sensitivity to amplitude rise times (ARTs or speech “edges”) in the envelope, accompanied by less accurate neural encoding of low-frequency envelope information. Accordingly, enhancing these characteristics of the speech envelope may enhance neural speech processing in children with dyslexia. Here we applied an envelope modulation enhancement (EME) algorithm to a 10-min story read in child-directed speech (CDS), enhancing ARTs and also enhancing low-frequency envelope information. We compared neural speech processing (as measured using MEG) for the EME story with the same story read in natural CDS for 9-year-old children with and without dyslexia. The EME story affected neural processing in the power domain for children with dyslexia, particularly in the delta band (0.5–4 Hz) in the superior temporal gyrus. This may suggest that prolonged experience with EME speech could ameliorate some of the impairments shown in natural speech processing by children with dyslexia.

## Introduction

Even after decades of research, the neurophysiological origins of developmental dyslexia remain debated. Although the accepted hallmark of developmental dyslexia at the cognitive level is the “phonological core deficit” (Stanovich, 1988; defined by children’s ability to identify and manipulate phonological units like syllables and phonemes in oral tasks), the neurophysiological basis of these phonological difficulties is not well-understood. Here, we adopt the sensory-neural Temporal Sampling Framework (TSF, Goswami, 2011, 2015; see Goswami, 2022 for a recent review) to explain the aural difficulties found in children with developmental dyslexia and potentially devise sensory-neural modes of remediation. The TSF takes a neuro-oscillatory perspective to phonological development, proposing that the phonological difficulties that characterise children with dyslexia may arise because of inaccurate synchrony between speech rhythms [particularly those dependent on amplitude envelope information in low-frequency bands of AMs focussed on ∼2 and ∼5 Hz, see Leong and Goswami (2015)] and ongoing neural oscillations. The TSF assumes that less accurate speech-brain alignment is related to impaired discrimination of amplitude rise times (ARTs) by children with dyslexia. These ART impairments have been shown in studies in 7 different languages [English, French, Finnish, Hungarian, Dutch, Chinese and Spanish: see Goswami (2015), for a review]. ARTs are known from adult studies to phase-reset ongoing cortical oscillations in different electrophysiological frequency bands such as delta (0.5–4 Hz), theta (4–8 Hz), and gamma (25–40 Hz). For example, Gross et al. (2013), have shown using MEG that ARTs (termed “speech edges”) phase-reset ongoing neural oscillations in the adult auditory cortex—particularly in the delta and theta band—thereby improving speech-brain alignment.

Indeed, it has been shown experimentally that speech edges serve a critical function as acoustic landmarks which aid the segmentation of incoming speech information (Doelling et al., 2014). The TSF postulates that the identification of ARTs and related oscillatory phase alignment to AM information at lower frequencies (slow speech rhythms <10 Hz) is atypical in children with dyslexia. Regarding the sensory aspects of TS theory, impaired ART discrimination has recently been documented in pre-verbal infants at family risk (FR) for dyslexia (Kalashnikova et al., 2018), and individual differences in sensitivity to ART predict vocabulary development by age 3 years (Kalashnikova et al., 2019). Further, ART has been related to phonological development in the preschool years in several longitudinal studies (Corriveau et al., 2010; Law et al., 2017; Vanvooren et al., 2017). Accordingly, the data suggest that ART discrimination is impaired in children at FR for dyslexia long before they begin learning to read, with negative consequences regarding their phonological development.

Meanwhile, the neural side of the TSF has been supported by a growing body of neurophysiological studies of speech processing conducted with children with dyslexia (Molinaro et al., 2016; Power et al., 2016; Di Liberto et al., 2018; Destoky et al., 2020, 2022; Mandke et al., 2022). In all these studies, which cover a range of languages, cortical tracking of speech was selectively impaired in frequency bands below 10 Hz, i.e., dyslexics showed decreased synchronisation with the speech stimulus in the delta band. Molinaro et al. (2016, Spanish, MEG) reported cortical tracking impairments in dyslexia in the 0.5–1 Hz range, whereas Power et al. (2016, English, EEG) reported cortical tracking impairments in dyslexia in the 0–2 Hz range, both within the canonical delta band (0.5–4 Hz). Destoky et al. (2020, French, MEG) and Di Liberto et al. (2018, English, EEG) reported impaired cortical tracking in a broader low-frequency band below 8 Hz. Finally, Destoky et al. (2022) reported impaired cortical tracking in dyslexia in different speech-in-noise conditions at both a “phrasal”’ rate (0.2–1.5 Hz) and a “syllable” rate (2–8 Hz), depending on the noise condition. These phrasal and syllable rates were not determined by analysis of the low-frequency envelope information in the story input, rather they were assumed by the experimenters. In Mandke et al. (2022, English, MEG) both stress and syllable rates were determined by modelling the speech input that formed the stimulus during the MEG recordings. Mandke et al. (2022) reported speech-tracking deficits in dyslexic children at both the stress/prosodic rate (0.9–2.5 Hz) and the syllable rate (2.5–5 Hz) for their stimuli. Convergent data come from oscillatory studies of the tracking of AM noise, a stimulus designed to mimic the amplitude envelope of the speech signal. Both children with dyslexia (Peter et al., 2023, EEG) and adults with dyslexia (Hämäläinen et al., 2012, MEG) demonstrated a selective difficulty in the cortical tracking of AM noise modulated at 2 Hz, difficulties that were not present at other AM frequencies (children: 5, 8 Hz; adults: 4, 6, 8, 10 Hz). In an AM noise study using fNIRS (Cutini et al., 2016), right hemisphere responses (HbO concentration) to 2 Hz modulation differed between children with and without dyslexia. However, responses to 40 Hz AM did not differ by group nor by hemisphere. Interestingly, individual differences in ART sensitivity were significantly related to the HbO differences in the children when the stimulus was 2 Hz modulated. It is worth noting that studies of cortical speech tracking in adults with dyslexia have not yielded consistent low-frequency deficits. For example, a study of adults with dyslexia reported by Lizarazu et al. (2021b) could not replicate group differences typically reported in the delta and theta bands. In another adult study by Thiede et al. (2020), decreased synchronisation for the participants with dyslexia in the delta and high gamma bands was reported in addition to increased synchronisation in the theta, beta, and low gamma bands.

**FIGURE 1:**
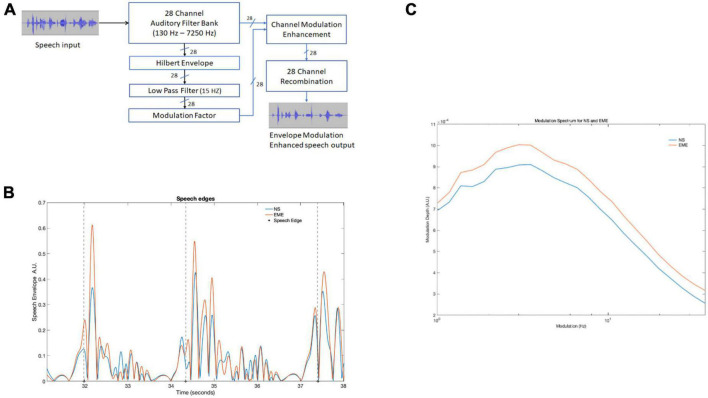
**(A)** Depicts the entire processing pipeline involved in the speech envelope modulation enhancement algorithm. **(B)** Three edge onsets (marked by dotted lines) identified by the algorithm in both natural and EE speech conditions in a 7-s speech recording. The large peaks after the dashed line were identified by the algorithm. **(C)** Shows modulation spectrum of the entire story for the two conditions (NS and EME). The figure depicts EME as having higher modulation. NS, natural speech; EME, envelope modulation enhancement.

The neurophysiological studies reviewed above are important, as they utilised continuous listening tasks with ecological validity (such as sentence listening or story listening). As noted, efficient synchronisation between speech rhythms (amplitude envelopes) and neural oscillations relies on the accurate processing of large amplitude landmarks (ARTs) in the speech envelopes, frequently termed speech edges. Speech edges are transient events marked by rapid onset and large amplitude (as shown in Figure 1B). Given the impairments in ART discrimination in dyslexia that are found across languages, it could be expected that individuals with dyslexia have impaired neural phase-resetting mechanisms, which may contribute to the impairments seen in cortical speech tracking. This hypothesis has only previously been tested in adults. Lizarazu et al. (2021a) investigated the phase-resetting mechanism in adults with and without dyslexia (French speakers) using MEG. They computed phase locking values (PLVs) between neural oscillations and the speech envelope time-locked to edge onsets. Their key finding was that adult participants with dyslexia showed significantly weaker PLVs compared to control participants. The locus of reduced PLVs was the left auditory cortex. This contrasts with the cortical tracking of speech studies involving children reviewed above, which have typically identified right hemisphere differences in atypical cortical speech tracking in dyslexia. However, in cortical tracking studies testing adults with dyslexia, left-hemisphere rather than right-hemisphere differences have been reported (Lehongre et al., 2013). Accordingly, hemisphere differences in speech encoding studies that include participants with dyslexia may reflect developmental effects such as ageing and/or the effects of increased reading experience (see section “Discussion” in Cutini et al., 2016).

Regarding the remediation of dyslexia, the available neurophysiological data regarding speech edges and cortical tracking suggest that enhancing speech edge information in natural speech could facilitate accurate cortical tracking by participants with dyslexia. This question was first explored by Van Hirtum et al. (2019). In a study with dyslexic adults who spoke Dutch, Van Hirtum et al. (2019) implemented a speech envelope enhancement (EE) algorithm drawn from cochlear implant research, which exaggerated the onset cues (ARTs) of a given speech envelope for a sentence. They reported an improvement in speech reception thresholds (SRTs) for the dyslexic participants in both speech-in-noise and noise-vocoded conditions. The average SRT difference between EE and non-EE speech for the speech-in-noise condition was 0.55 dB for controls and 0.84 dB for dyslexic participants. In the vocoded speech condition, the average difference was 0.60 dB for the controls and 0.61 dB for dyslexic participants. A version of the adult study, using the same speech material, was then carried out with children (Van Hirtum et al., 2021). Significant differences were again found. The average difference in SRT between EE and non-EE for natural speech was 0.59 dB for the control children and 0.49 dB for dyslexic participants, while the average difference in SRT between EE and non-EE for noise-vocoded speech was −0.38 dB for controls and −0.41 dB for dyslexics. Whereas the typical test–retest reliability of SRT is typically around 1.1–2.6 dB for adult listeners (Plomp and Mimpen, 1979; Bentler, 2000; Luts et al., 2008; Jansen et al., 2012), it is worth noting that the test–retest reliability is influenced by the speech material. The measurement error reported by Van Hirtum et al. (2019) is 0.72 dB. Hence, all but one of the eight significant findings are smaller than the measurement error, suggesting that these significant effects below 0.72 dB could reflect noise in the experiment (Anvari and Lakens, 2021).

Nevertheless, all the changes in threshold in the Dutch studies were in the expected direction, and the data are consistent with the TSF, which would predict that enhancing ARTs should enhance speech processing for individuals with dyslexia. Whether the instantaneous enhancements documented by Van Hirtum et al. (2019) led to gains in phonological awareness for their participants was not studied.

To examine this transfer question developmentally, longitudinal studies are required. Van Herck et al. (2022) designed a developmental study of EE with Flemish-speaking pre-reading children who were at cognitive risk (CR) for dyslexia. The EE algorithm developed by Van Hirtum et al. (2021) for adults was applied to natural child-directed speech and presented as stories on a tablet computer. The children also played GraphoGame in Flemish, a computerised software for teaching phonological awareness and phonic knowledge (letter-sound correspondences). The intervention was carried out in children’s homes by their parents, who were asked to play the games and listen to the stories with their children daily. Following 12 weeks of intervention, the group of CR pre-readers receiving the EE stories showed a significant improvement in ART discrimination thresholds compared to the CR children who listened to the stories without EE. This was an interesting observation, as the improvement occurred at a critical period in development, namely before reading instruction had begun. In a second assessment of the same children, Vanden Bempt et al. (2022), investigated potential effects on phonological awareness and reading-related skills. Vanden Bempt et al. (2022) reported no differences between the two groups for phonological awareness and reading-related skills. As both groups of CR children had played GraphoGame Flemish, however, both the EE and non-EE groups were receiving an intervention designed to teach phonological awareness and reading-related skills. Thus, the additional intervention GraphoGame Flemish could explain the lack of an effect of EE between the two CR groups. Accordingly, it remains unclear whether the improvements in speech-in-noise perception demonstrated by Van Hirtum et al. (2019, 2021) or the improvements in rise time discrimination reported by Van Herck et al. (2022) can ameliorate the phonological processing difficulties that characterise individuals with dyslexia and thereby affect reading.

In the current study, we aim to build on the results of our existing spectral analyses of the temporal modulation structure of English child and infant directed speech (S-AMPH modelling; Leong and Goswami, 2015) along with our previous behavioural findings regarding the effects of the acoustic structure of natural speech on phonological processing for children with and without dyslexia (Flanagan and Goswami, 2018), to ameliorate the low-frequency speech-brain synchronisation difficulties proposed by TS theory. Our approach was based on an oscillatory-temporal sampling perspective which, in addition to enhancing ARTs, was designed to selectively amplify low frequency (<10 Hz) modulation in the speech envelope. We term this an Envelope Modulation Enhancement (EME) algorithm in order to distinguish it from the EE algorithm used in the Flemish studies. In the current study, we investigated whether EME speech would change instantaneous neural speech encoding for children with dyslexia. Children with and without dyslexia aged ∼9 years listened to a story spoken in natural child-directed speech (CDS) and then to an EME version of the same story while their neuromagnetic (MEG) activity was recorded. *A priori* we expected that the children with dyslexia (relative to controls) would show improved instantaneous responses to EME speech edges, specifically in the delta band (0.5–4 Hz). As the same participants had also listened to parts of the story in natural speech (NS) prior to receiving the EME speech (Mandke et al., 2022), and the children with dyslexia had shown reduced neural synchronisation to NS in the delta band which was right-lateralised, we also predicted *a priori* that any neural changes with EME speech would be right-lateralised. The original study design was that following pre-testing with EME speech, the children with dyslexia would receive a 9-week intervention with EME speech, following which post-intervention imaging would take place. This experimental design could not be fulfilled due to the COVID-19 Pandemic. Accordingly, we present only the pre-intervention data exploring potential instantaneous effects of EME speech in the current manuscript.

## Methods

### Participants

Thirty-nine participants took part in the present study, which is part of an ongoing longitudinal study of dyslexia (2018–2023). These children had previously participated in Mandke et al. (2022). In that study only natural speech MEG data were analysed. Reduced neural lagged coherence for low-frequency speech information in auditory and fronto-temporal regions in children with dyslexia was reported. Children were aged on average 7–8 years when the study began (2018). The current sample comprised all those children from the total cohort of 121 children who volunteered for neuroimaging (two further control volunteers were excluded as they were significantly younger). We report MEG data from 20 age-matched controls (CA group) and a group of 19 dyslexic children (DY group), see Table 1 for details. Due to interruptions to the research caused by COVID-19, the CA volunteers were slightly younger than the DY group once data collection was completed, even though these groups are well-matched for age in the larger cohort. Some dyslexic children were also pre-tested during the subsequent school year, when we had planned a second EME intervention; again, the COVID-19 Pandemic prevented this research design from being implemented. The inclusion criteria for children with dyslexia were that the child had English as their first language, IQ in the average range, and scores at least 1 SD below the mean on at least 2 of the 4 tests of reading and spelling (see Supplementary material). For age matched controls, the inclusion criteria were that children have English as their first language and have average attainment in reading and spelling for their age. Children with dyslexia were recruited via Special Education Needs co-ordinators, and only children who had no additional learning difficulties [e.g., dyspraxia, ADHD, autistic spectrum disorder, developmental language disorder (DLD)], a non-verbal IQ above 84, and English as the first language spoken at home were included. The absence of additional learning difficulties was based on school and parental reports and our own testing. Participants were attending state schools (equivalent to US public schools) situated in a range of towns and villages near a university town in the United Kingdom. All children received a short hearing screen using an audiometer. Sounds were presented in both the left or right ear at a range of frequencies (250, 500, 1000, 2000, 4000, 8000 Hz), and all children were sensitive to sounds within the 20 dB HL range. All participants and their guardians gave informed consent in accordance with the Declaration of Helsinki, and the study was reviewed by the Psychology Research Ethics Committee of the University of Cambridge, UK. The data presented in this study were acquired in the summer 2019 (*N* = 32 recordings) and autumn of 2020, as data collection was impacted by the COVID-19 pandemic. Data from 7 of the 19 DYs was acquired in 2020 by following appropriate University-approved COVID-19 safety protocols. Although these children were in a higher school year than the other dyslexic children, some of the CA children tested previously had also been in a higher school year, hence this was not expected to exert any systematic effects on the data.

**TABLE 1:** Group performance on psychoacoustic and standardised cognitive, language and literacy measures.

	Control group (*N* = 20)	Dyslexic group (*N* = 19)
Age (years)*	8.81 (±0.60)	9.66 (±0.77)
Gender	M: 15, F: 5	M: 10, F: 9
FSIQ	103.79 (±11.12)	107.25 (±11.35)
TOWRE word*	105.75 (±7.15)	79.05 (±11.28)
TOWRE non-word*	95.50 (±11.36)	73.32 (±11.46)
BPVS3*	104.70 (±11.35)	101.05 (±13.14)
BAS spelling*	98.10 (±6.91)	81.58 (±4)
BAS reading*	97.95 (±3.85)	83.26 (±5.87)
Rise time sensitivity (ms)*	80.46 (±38.46)	110.16 (±34.01)

*Values significantly different between groups (*p* < 0.05, 2-tailed *t*-test). A full description of these measures can be found in Supplementary material.

### Speech stimuli

The participants were presented with a 10-min audio recording of the *Iron Man* story read by a female native British English speaker. The stimuli were recorded digitally using an AKG© C1000S cardioid microphone onto a Tascam© DR-100 digital recorder at a sampling rate of 48 kHz.

### Envelope modulation enhancement

The modulations in the original speech stimuli were enhanced using the following approach (see Figure 1A). The 10-min recording of speech was divided into 10 one-min sections. Each section contained a whole phrase/utterance and included 500 ms of silence at the beginning and end to avoid edge effects. The sound file was reduced to mono from stereo, with the sampling rate kept at the original rate of 48 kHz. Each sound file was filtered by a 28-channel filter bank spanning 100 Hz to 7250 Hz. The filter bank was designed with adjacent overlapping Finite Impulse Response (FIR) bandpass filters, with ERB_N_ spacing (see Supplementary Table 1 for filter edge frequencies). The filter bank design models the cochlear channels in a normal-hearing individual at moderate levels (Stone and Moore, 2003). Channel delays introduced by the filters were compensated for. See Supplementary Figure 1 for filter bank frequency responses. For each sound file, 28 Hilbert envelopes were generated, one for each of the 28 spectral channels. To extract the modulations of interest (i.e., up to 10 Hz) the Hilbert envelopes were low pass filtered using a zero-delay recursive MATLAB *smooth* function. This digital moving average filter had a −6 dB cut-off frequency at 15 Hz, to ensure the modulations of interest in the canonical ranges 0.5 to 4 Hz (delta) and 4 to 8 Hz (theta) was well within the −3 dB passband. The low-pass filtered Hilbert envelopes were then processed to form the envelope modulation enhancement (EME). A modulation factor (MF) was given to each spectral channel. The MF was calculated as the ratio of each channel’s RMS to the maximum RMS channel value. This results in the most modulated channel having a modulation factor of 1, and all other channels having a value at or below 1. Based on the modulation factor, a dynamic range compressed gain factor was then applied to each envelope. If the channel modulation factor was below 0.1, a fixed amplification of 20 dB was applied. For values between 0.1 and 1, the gain was linearly reduced so that 0 dB gain was applied for a modulation factor of 1. To avoid the amplification of noise, amplification was restricted to levels greater than −60 dB. The resulting enhanced envelopes were multiplied by their corresponding original spectral channels. The EME signal was reconstructed from the sum of the EME channels. The EME sound files were equated for loudness with their non-enhanced sound files using a perceptual loudness model (Moore et al., 2016). Perceptually, however, this had the effect of making the EME speech sound slightly quieter than the natural speech.

### Speech stimuli, procedure

At the start of every MEG session, a 5-min resting-state scan was recorded with children in a seated position with their eyes open. Participants were asked to relax and focus on a fixation cross while avoiding any excessive movements or eye blinks. After the resting-state scan, participants listened to a 10-min recording of the children’s book The Iron Man: A Children’s Story in Five Nights by Ted Hughes (NS). They subsequently also listened to the same 10 min of the story presented as EME speech (i.e., in the same listening session), and 10 min of reversed unprocessed speech. The rationale for using the same 10-min story recording as both NS and EME speech was to ensure identical acoustic characteristics between conditions except for the application of the EME. In this manuscript, the data from the naturalistic listening condition and EE speech condition are reported. The EME speech was always heard second. For details regarding natural speech story listening and resting-state data, we direct the reader to Mandke et al. (2022). The recording was presented diotically using magnetically safe foam-tipped insert earphones (ER-1, Etymotic Research) at a comfortable listening level (approximately 70 dB SPL). The speech material was divided into 5 trials of 2 min. Each trial began with a 1-s-long calibration tone (500 Hz) followed by 2 s of silence. The stimuli were presented using NBS Presentation software.^1^ At the end of a trial, the experimenter spoke to the participants over the intercom, asking them to adjust their head position to match their starting position and checking their comprehension of the story by asking simple questions **(e.g., What was the animal in the story?, At the end, what colour was the eye?)**. This step ensured we acquired data with minimal head movement. Trials with >8 mm head movement were rejected from any further analysis. The participants’ responses to the oral story-related questions indicated good comprehension, accordingly these responses were not saved and analysed. Excessive head movements were most common toward the end of the experiment when the children listened to the reversed speech. However, those data are not reported here.

### Data acquisition

The data were acquired using a 306-channel VectorView (Elekta Neuromag) system at the Cognition and Brain Sciences Unit, Cambridge (CBSU). The scanner has 1 magnetometer and 2 orthogonal planar gradiometers at each of the 102 locations. During the course of the study, the MEG scanner at CBSU was upgraded to Elekta Neuromag Triux Neo in January 2020. This offers the same sensor configuration as VectorView. All other acquisition parameters were kept constant between the two scanners and are as follows –

Participants were seated in a magnetically shielded room. The data were sampled at 1000 Hz, and bandpass filtered at 0.03–330 Hz. For all the participants we recorded continuous head position throughout each run using 5 head position indicator (HPI) coils fixed to the head. Prior to data acquisition, we used Polhemus Isotrak to digitise the locations of fiducial markers (the nasion, left and right pre-auricular points), the HPI coils, and a number of additional head points. This information was used to perform accurate co-registration of MEG and MRI data. Additionally, we also acquired vertical, horizontal electro-oculograms (V-EOG, H-EOG) and electro-cardiogram (ECG), which were used for artefact rejection. Data from 7 of the 19 children with dyslexia was acquired on the Triux Neo scanner, whereas remaining MEG data was acquired on the VectorView system.

### MEG data pre-processing

In the offline analysis, all the data were subjected to temporal signal space separation (tSSS) method to remove external noise and the head movements were compensated using MaxMove software, both as implemented in MaxFilter version 2.1 (Elekta Neuromag). All the analyses were performed using Brainstorm (Tadel et al., 2011) in Matlab 2020b (Mathworks). Brainstorm is documented and freely available for download under GNU general public licence.^2^ The continuous MEG data were down-sampled to 250 Hz, and bandpass filtered between 0.5 and 48 Hz. We used the data from V-EOG, H-EOG, and ECG to mark artffacts in the MEG data. Signal space projections (SSPs) were calculated automatically to remove magnetic interference created by eyeblinks, eye movements and heartbeats (Uusitalo and Ilmoniemi, 1997). SSP, unlike other data cleaning methods, relies on the fact that electromagnetic fields generated by sources originating from outside the brain (e.g., eye blinks, heartbeats) have spatial distributions that are different from neural sources. The SSP algorithm concatenates all the artefact events and performs a singular value decomposition. Singular vectors with the highest singular values are selected and their projection is subtracted from the MEG data. For offline analysis, 10 min recording per condition were split into 60-s epochs with 2-s pre- stimulus intervals. Data segments were manually examined to identify any excessive head movements, sensor jumps or muscle artefacts. Such segments were marked and excluded from further analyses. Following these steps, all participants yielded 10 min of MEG data per condition, with the exception of one participant from the CA group (6 min) and one participant from the DY group (8 min) for both the conditions.

### Data analysis

#### Identification of speech edges

Speech edges in the ten 1-min envelope modulation enhancement (EME) continuous stimuli were identified using a thresholding algorithm similar to the one used by Gross et al. (2013). The stimuli were first down sampled to 1 kHz, and the wideband Hilbert envelopes were computed. Each Hilbert envelope was then down-sampled to 100 Hz and amplitude scaled to be between 0 and 1. The algorithm searched for three conditions: (1) a pre-edge-onset period of low signal (mean amplitude less than 0.05) of at least 400 ms long, (2) followed by a post-onset period of higher signal (mean amplitude greater than 0.05) of at least 1000 ms long, and (3) the mean difference in level in the 20 ms immediately before and after the onset to be greater than 0.05. Each epoch containing an onset was 1400 ms long. Illustrative examples of speech edges are shown in Figure 1. Onsets were verified by visual inspection and epochs that overlapped were not counted. A total of 131 speech onsets were identified and marked both in the natural speech (NS) and EME speech conditions.

We also quantified the phase locking value. However, we could not replicate previously reported effects in the literature with dyslexic adults (Lizarazu et al., 2021a) and non-dyslexic adults (Gross et al., 2013).

#### MEG source analysis

For the 32 of the 39 children who consented to MRI scanning, we co-registered their MEG data to the child’s T1-weighted structural MRI image acquired using a 3T Siemens Tim Trio and an MPRAGE sequence. For subsequent source analyses, the nasion and left and right pre-auricular points were first marked manually in each participant’s MRI volume. These were used as starting points for the co-registration of MEG-MRI data, which was improved using an iterative closest point algorithm as implemented in Brainstorm. For the children who opted out of an MRI scan (*N* = 7, DY) we used a standard Montreal Neurological Institute (MNI) template. We used their digitised head shape to warp the standard anatomy to create “pseudo-individual” anatomy. The “warp” option in Brainstorm deforms the MRI and all the surfaces of the template to match the head shape defined using digitised head points.

The scalp and all the cortical surfaces were extracted from the MRI volume using FreeSurfer (Fischl, 2012). A cortical surface triangulation was obtained using the “recon-all” pipeline with default parameters and was imported into Brainstorm. The high-resolution cortical surfaces of all the participants were down-sampled to 15,000 triangle vertices for source analysis. Forward model was based on the overlapping spheres method (Huang et al., 1999). The noise covariance was estimated using a 2-min empty room recording acquired at the start of every session. Data covariance was estimated using the 2-s baseline period before every epoch. The speech edges were marked on the MEG sensor level data and the MEG data were epoched time-locked to the speech edges. Each epoch included a 400 ms pre-stimulus (or pre-edge-onset) interval and a 1000 ms post-stimulus (or post-edge-onset) interval. These epochs were source-localised using a Linearly Constrained Minimum Variance Beamformer (LCMV) (Van Veen et al., 1997). The beamformer combined information from both the magnetometers and planar gradiometers. The LCMV beamformer constructs a set of spatial filters that are applied to the sensor data to reconstruct the signal at every given vertex of the cortical surface while minimising the variance or contributions from all other locations. The process is repeated across the whole brain space to achieve a whole-brain source reconstruction. The source analysis was performed for both NS and EME speech conditions.

#### Time domain analysis (ERP)

The 131 edge onsets were marked as epochs/trials per participant per condition (NS, EME speech) and were further source localised. To reduce the high number of trials, epochs within a 60 s time window were averaged in source space to arrive at 10 averaged source level time series per participant per condition (for 10 min of listening). The averaged trials were z-transformed. Further, absolute values were extracted, and the time courses were projected onto Montreal Neurological Institute (MNI) template brain using a non-linear transformation. The data were spatially smoothed using a Gaussian kernel of 8 mm (FWHM). These steps were carried out to measure the phase locked responses to edge onsets in our data.

#### Phase locking value (PLV)

We computed the PLV to measure the phase synchronisation between source localised trials *x*(t) and the corresponding speech envelope *y*(t) time-locked to speech edges. The procedure was the same as the one reported by Lizarazu et al. (2021a). Here, we filtered both the source time series and speech envelope between 0 and 8 Hz to measure phase locking in the delta and theta band. Phase locking value (PLV) measures frequency-specific phase synchronisation between two signals. It was computed by calculating the distribution of phase difference extracted from two source time series *x*(t) and *y*(t).

It is formally given by –


(1) $$P\,L\,V_{t}=\frac{1}{N}\,\left|\sum_{n=1}^{N}\text{exp}\,\left(j\,\theta \,\left(t,n\right)\right)\right|$$

Where, θ(*t*, *n*) gives the phase difference ϕ_1_(*t*, *n*) − ϕ_2_(*t*, *n*). PLV provides a summary statistic of the phase difference at *t*. The phase information was extracted using Hilbert Transform.

The PLV between the two signals was calculated for each time point from −400 ms (pre-stimulus interval) to 1000 ms (post-stimulus interval). These PLVs were then averaged across time and projected onto Montreal Neurological Institute (MNI) template brain using a non-linear transformation (as implemented in Brainstorm). The data were spatially smoothed using a Gaussian kernel of 8 mm (FWHM). These steps were repeated for every participant to obtain a source-level map of PLV. These individual maps were subjected to non-parametric permutation testing as described in the statistical analysis section below.

#### Spectral analysis

Spectral power was then estimated for the significant ROIs in low-frequency bands, namely from the bilateral STG in the delta (0–4 Hz) and theta (4–8 Hz) band between groups (CA vs. DY) and between conditions (NS vs. EME). We averaged activity in these spectral bands in the post-stimulus interval. Lastly, we estimated the theta-delta ratio by dividing the averaged band power in the theta band by the averaged band power in the delta band, per epoch. This was of interest because recent dyslexia modelling data (Araújo et al., 2022) and infant language acquisition data (Attaheri et al., 2022) from our group has indicated that worse language outcomes are associated with a higher theta-delta ratio.

#### Statistical analysis

To identify cortical regions of interest (ROIs) showing statistically significant activity, a one-tailed student’s *t*-test was performed on the ERP data. Here, post-stimulus intervals were compared against the baseline for both NS and EME speech in CAs and DYs.

To test between-group effects for the ERP and PLV data, a non-parametric permutations test was used. For all the between-group comparisons permutations tests were performed across subjects for random effects inference. For the null hypothesis of no difference between groups (DYs vs. CA) or conditions (NS vs. EME speech), the group labels were randomly permuted. The subsequent data-driven distribution was used to compute a *t*-test statistic in source space. The permutations procedure was repeated 5000 times using a Monte Carlo method, allowing us to compute a t-statistic at every vertex in the source space, thus converting the raw values into *p*-values. False discovery rate (FDR) was applied to correct for multiple comparisons (Benjamini and Hochberg, 1995). To test for potential effects of EME, we used a repeated measures 2 × 2 × 2 × 2 analysis of variance (ANOVA) with three within-subject factors: speech (NS, EME), frequency band (delta, theta), and hemisphere (left, right), with group as the between-subject variable (DYs, CAs). To correct for multiple comparisons, a factor or an interaction effect was treated as statistically significant at *p* < 0.005 (Bonferroni’s correction). All the spectral data were examined for outliers. These were defined as values greater than 3 scaled median absolute deviations, which were further replaced by performing a linear interpolation as implemented in Matlab (filloutliers, method: linear).

## Results

### Time domain analysis (ERP)

**FIGURE 2:**
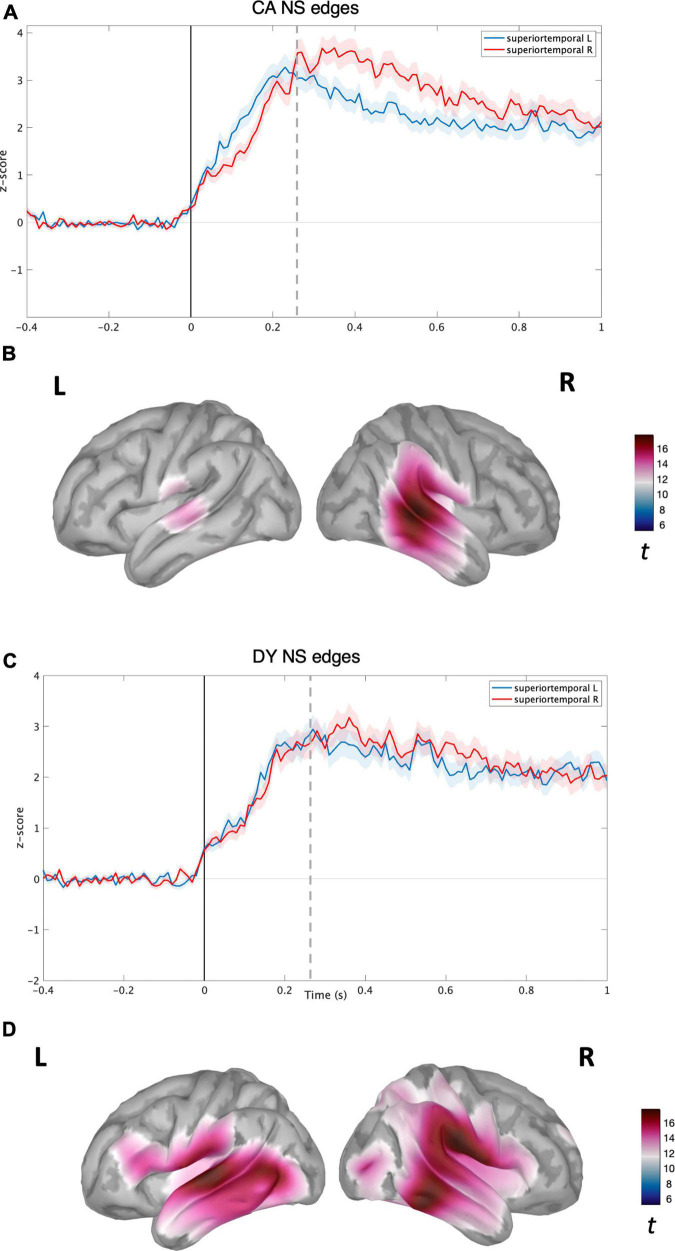
Source level time-domain within-sample analysis for NS condition. **(A)** Averaged time course obtained from bilateral superior temporal gyrus for NS condition with standard error (shaded) plotted on top (Z-score) for CAs. **(B)** Left and right cortical maps depicting statistically significant (*p* < 0.05, FDR) activity (post-stimulus >pre-stimulus) for NS condition (*t*-values). **(C)** Averaged time course obtained from bilateral superior temporal gyrus for NS speech condition with standard error (shaded) plotted on top (z-score) for DYs. **(D)** Left and right cortical maps depicting statistically significant (*p* < 0.05, FDR) activity (post-stimulus >pre-stimulus) for EME speech condition (*t*-values). The dotted line corresponds to the time point (270 ms) plotted in brain space.

**FIGURE 3:**
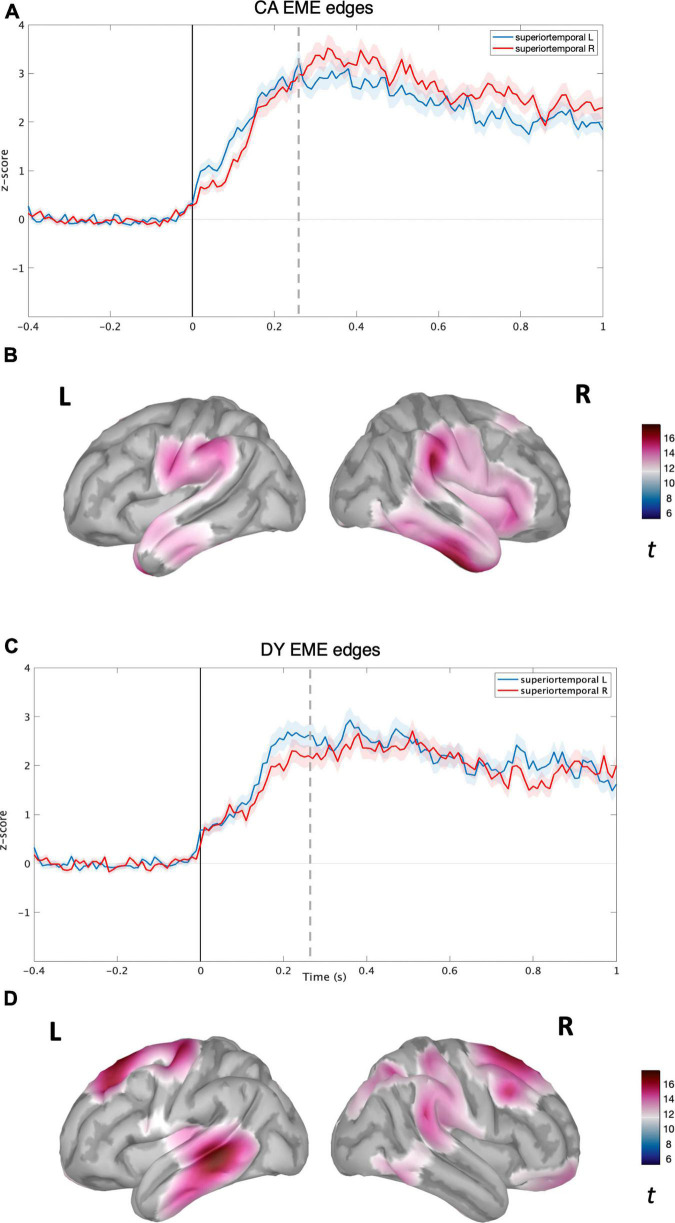
Source level time-domain within-sample analysis for EME condition. **(A)** Averaged time course obtained from bilateral superior temporal gyrus for EME condition with standard error (shaded) plotted on top (z-score) for CAs. **(B)** Left and right cortical maps depicting statistically significant (*p* < 0.05, FDR) activity (post-stimulus >pre-stimulus) for NS condition (*t*-value). **(C)** Averaged time course obtained from bilateral superior temporal gyrus for EME speech condition with standard error (shaded) plotted on top (z-scores) for DYs. **(D)** Left and right cortical maps depicting statistically significant (*p* < 0.05, FDR) activity (post-stimulus >pre-stimulus) for EME speech condition. The dotted line corresponds to the time point (270 ms) plotted in brain space.

Data from all the participants was used in the analysis (CA = 20, DY = 19). The averaged time courses per condition per participant were visualised. This allowed us to identify one participants’ data for one condition (DY EME) as having values orders of magnitude higher than the group average. As a result, these data was removed from further analysis. To identify significant ROIs, we performed a time domain analysis using the 131 speech edges in each condition (NS and EME) as described above. A one-tailed *t*-test compared the post-stimulus interval (0 to 1000 ms) against the pre-stimulus baseline (−400 to 0 ms). The data presented in Figures 2, 3 are within-sample comparisons (*p* < 0.05, FDR).

For the CA group in the NS condition, activity was localised in the bilateral temporal regions (see Figure 2B) with the maxima located in the right superior and middle temporal regions. The time courses of activity from bilateral superior temporal regions are shown in Figure 2A. For the CA group in the EME condition, the neural activity was much more diffuse (see Figure 2D), extending to the right hemisphere’s middle and inferior frontal gyrus regions, and inferior motor regions in the left hemisphere. The reconstructed time courses from bilateral superior temporal gyri were much more similar (see Figure 2C) compared to the NS condition (Figure 2A).

For the DY group in the NS condition, the speech edge evoked activity was predominantly localised in the bilateral auditory belt area, superior, middle, and inferior temporal lobes, further extending to IFG, MFG, and the inferior part of the motor area (see Figure 3A). The time course of activity generated from the bilateral STG regions also appeared highly similar, without the right hemisphere lateralisation shown for the CA group (see Figure 3B). In the DY group for the EME condition, the source level activity was sparse, with a peak in the left superior and middle temporal regions (see Figure 3D). The activity again further extended to non-auditory regions such as pre-frontal and motor regions. The source-level time course of activity for the DYs in the EME condition thus appeared similar to the NS condition, with activity mainly in the bilateral STG and further extending to non-auditory areas such as motor and pre-frontal regions. While the amplitude of the overall response was slightly lower than for natural speech, there was a shift toward the left STG (see Figure 3C).

Following this analysis, we performed several group comparisons at both the whole brain level and by using bilateral STGs as ROIs to check for group differences in the temporal domain. We utilised a non-parametric permutations test (*p* < 0.05, FDR) to test these hypotheses across time points. First, we compared NS and EME condition data within each group, i.e., DY NS vs. DY EME, and CA NS vs. CA EME. This was found to be non-significant (*p* < 0.05, FDR). Second, we compared NS and EME condition data between the two groups, i.e., CA vs. DY (NS), CA vs. DY (EME). These comparisons were also found to be non-significant (*p* < 0.05, FDR). Accordingly, in the time domain, any differences between groups that appear present when comparing Figures 2, 3 were all non-significant.

### PLV

To assess whether the groups differed in the phase domain, we performed a series of statistical comparisons in the phase domain viz., (1) NS CA vs. EME CA, (2) NS DY vs. EME DY, (3) NS CA vs. NS DY, and (4) EME CA vs.. EME DY. However, none of these statistical comparisons yielded a statistically significant finding. As a result, we could not replicate the left lateralised delta and theta band group difference (between controls and dyslexics) reported by Lizarazu et al. (2021a). Increased phase locking following speech edge onset has also been reported in healthy listeners by Gross et al. (2013). It is worth noting that both these studies had adult participants whereas our study was with children. Younger participants often yield noisier data, e.g., due to excessive head movement, to the detriment of phase locking estimates.

### Spectral analysis

To test for the effects of EME in the spectral domain, delta and theta band power (averaged post-stimulus activity) were investigated. The reconstructed time courses from bilateral STG were Hilbert-transformed to extract spectral power in the delta (0–4 Hz) and theta (4–8 Hz) bands of interest. As noted, we had predicted that EME would selectively affect neural processing of speech by children with dyslexia in the delta band. A Kolmogorov-Smirnov test revealed that the data were normally distributed. Levene’s test of equality of error variances revealed that the data were homogenous for all levels of the repeated measures. And finally, Box’s test of equality of covariance matrices was not significant, indicating that the covariance was equal across groups. Accordingly, a repeated measures ANOVA as described earlier was run, using individual spectral values as the DV. We predicted a significant interaction between group, frequency band, and speech condition.

The results revealed that there was a significant main effect of both speech condition (NS vs. EME), *F*_(1,37)_ = 76.54, *p* < 0.001, η_p_^2^ = 0.674 and frequency band (delta vs. theta) *F*_(1,37)_ = 4896.06, *p* < 0.001, η_p_^2^ = 0.992. Spectral power was higher overall in both groups in the delta band than in the theta band, and spectral power was higher overall for the EME speech. There was also a significant main effect of group, *F*_(1,37)_ = 50.14, *p* < 0.001, η_p_^2^ = 0.580, because spectral power overall was lower in participants with dyslexia. The main effect of hemisphere (left vs. right) also reached significance, *F*_(1,37)_ = 6.197, *p* = 0.017, η_p_^2^ = 0.143, however, this effect did not survive the Bonferroni’s corrected threshold (*p* < 0.005). Regarding interaction effects, the predicted three-way interaction between speech condition, frequency band and group was significant, *F*_(1,37)_ = 417.63 *p* < 0.001, η_p_^2^ = 0.919, showing a large effect size. There were also significant interactions between speech condition and group *F*_(1,37)_ = 276.11, *p* < 0.001, η_p_^2^ = 0.882, and between frequency band and group *F*_(1,37)_ = 142.00, *p* < 0.001, η_p_^2^ = 0.793, again showing large effect sizes. The interaction between hemisphere and group was not significant following correction, *F*_(1,37)_ = 5.17, *p* = 0.029, η_p_^2^ = 0.123, and following correction nor were the interactions between speech condition, hemisphere and group [*F*_(1,37)_ = 5.46, *p* = 0.025, η_p_^2^ = 0.129], and frequency band, hemisphere and group [*F*_(1,37)_ = 6.50, *p* = 0.015, η_p_^2^ = 0.149]. All the remaining interaction effects were non-significant.

As the data were normally distributed, we performed *post-hoc* analyses using *t*-tests. Regarding the delta band, for the CA group the delta band responses in the left hemisphere (NS vs. EME) were not significantly different *t*(38) = 1.065, *p* = 0.293. However, in the right hemisphere the delta band responses were significantly different between NS and EME speech, *t*(38) = 2.232, *p* = 0.0316. In the DY group, when comparing NS and EME speech both the left hemisphere responses *t*(36) = −5.8503, *p* = 0.001 and the right hemisphere responses *t*(36) = −3.1704, *p* = 0.003 were significantly different. Regarding the theta band, for the CA group the responses in the left hemisphere were significantly different between conditions *t*(38) = 2.805, *p* = 0.007, whereas in the right hemisphere they were non-significant *t*(38) = 1.611, *p* = 0.115. Similarly, for the DY group in the left hemisphere the theta band responses were significantly different between conditions (NS vs. EME) *t*(36) = 2.338, *p* = 0.025 and the difference in the right hemisphere was non-significant *t*(36) = 1.934, *p* = 0.061.

**FIGURE 4:**
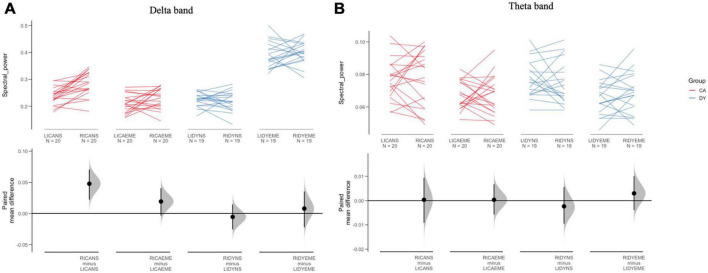
The paired mean difference for 4 comparisons are shown in the above Cumming estimation plot. The spectral responses for delta and theta band are plotted in panels **(A,B),** respectively. Spectral data are plotted on the upper axes; each paired set of observations (left, right hemisphere) is connected by a line. Each paired mean difference is plotted as a bootstrap sampling distribution on the lower axes. Mean differences are depicted as dots; 95% confidence intervals are indicated by the ends of the vertical error bars. LtCANS, left hemisphere CA natural speech; RtCANS, right hemisphere CA natural speech; LtCAEME, left hemisphere CA envelope enhanced; RtCAEME, right hemisphere CA envelope enhanced; LtDYNS, left DY natural speech; RtDYNS, right hemisphere DY natural speech; LtDYEME, left hemisphere DY envelope enhanced; RtDYEME, right hemisphere envelope enhanced.

*Post hoc* inspection of the significant 3-way interaction between speech condition, frequency band and group were carried out by using Cumming estimation plots (Ho et al., 2019; see Figure 4). As the hemisphere was of interest in the current study, and as some hemisphere effects were significant prior to correction, Figure 4 shows comparisons with the factor of hemisphere also included. In the Cumming estimation plot, the raw data, in this case, spectral power in the delta and theta bands, are plotted in the top panel. Each set of paired observations (right and left hemisphere) are connected by a line. The lower panels show a 95% confidence interval using vertical error bars and mean difference, plotted as dots (bootstrap sampling distribution). This method of data visualisation is common in the estimation statistics framework, allowing for a transparent way of visualising underlying treatment effects. When comparing the spectral power for the EME speech in the delta band with the spectral power for the non-EME speech in the delta band, there was a large increase in spectral power for the children with dyslexia in the EME speech condition and a slight reduction in spectral power for the control children. For the theta band, spectral power in both groups showed similar effects, reducing slightly in both groups for the EME speech. As can be seen from Figure 4, the EME speech reduced spectral power in comparison to natural speech for the CA group but increased spectral power for the DY group. In summary, the spectral analyses provide preliminary evidence that envelope-enhanced speech relative to natural speech leads to increased spectral power in the delta band for children with dyslexia.

The theta-delta power ratios were then computed for both the groups by condition and hemisphere and are shown in Table 2. The Kolmogorov-Smirnov test revealed that the data were normally distributed, hence *t*-tests were again used to investigate statistical differences. The theta-delta power ratio was not significantly different bilaterally in the control group for the NS (left–0.328, right–0.271) nor the EME (left–0.325, right–0.298) conditions. In the dyslexic group, however, the power ratios between the NS condition (left–0.346, right–0.345) and the EME condition (left–0.170, right–0.174) were significant (*p* < 0.0001). The EME speech reduced the theta-delta ratio for the children with dyslexia.

**TABLE 2:** Average theta-delta ratio between groups by conditions.

NS			EME	
	**Left hemisphere**	**Right hemisphere**	**Left hemisphere**	**Right hemisphere**
CA	0.328	0.271	0.325	0.298
DY	0.346***	0.345***	0.170***	0.174***

***p < 0.001.

## Discussion

Previous neuroimaging studies of natural speech listening by children with dyslexia have indicated impaired cortical tracking of the speech envelope in the delta band (Molinaro et al., 2016; Power et al., 2016; Di Liberto et al., 2018; Destoky et al., 2020, 2022; Mandke et al., 2022), and there also is developmental evidence for impaired discrimination of speech edges (ARTs) by children with dyslexia (Goswami, 2022). Accordingly, here we investigated whether enhancing speech envelope information in the delta and theta bands and enhancing speech edge information could change the neural processing of speech by children with dyslexia. We investigated these changes both in the time domain (ERP) and the phase domain (PLV). Against expectation, the ERP and phase domain analyses did not reveal any significant group or condition effects. However, we did find significant group effects in the spectral domain, with a significant group x frequency band x condition interaction, as predicted *a priori*. This shows that our implementation of the EME algorithm did indeed cause some instantaneous changes in how speech was processed by children with dyslexia. Delta band power changed in the EME condition in both groups, reducing in the control children in the right hemisphere and increasing for the children with dyslexia in both hemispheres. Theta band power changed for both groups, in the left hemisphere only, reducing with EME speech. The EME speech enhanced all modulations below 10 Hz, and this affected both delta and theta band processing in both groups.

The spectral changes related to hearing the EME speech found for the children with dyslexia showed large effect sizes. Accordingly, these spectral changes might reflect important mechanistic changes regarding neural speech processing following enhancement of the signal parameters of ART and low frequency envelope information. One potential interpretation relates to the theta-delta power ratio. We have previously modelled EEG data collected during natural speech listening by children with and without dyslexia, aiming to classify whether the listener has dyslexia or not based on the underlying neural dynamics identified by the TSF (Araújo et al., 2022). In that prior modelling with different participants (*N* = 48), the key parameter that identified a child as having dyslexia was a higher theta-delta power ratio during story listening (Araújo et al., 2022). As well as showing a mean higher theta-delta power ratio while listening to speech, the children with dyslexia also showed a higher variance in theta-delta ratio. Both effects were maximal across centrally located scalp areas. Individual differences in the mean power ratio were significantly negatively related to phonological awareness for the dyslexic children only (i.e., a higher ratio was associated with worse phonological awareness). In a related investigation of the TSF in infants, a higher theta-delta power ratio when listening to natural speech was found to predict *slower* language acquisition by infants (Attaheri et al., 2022, preprint). In their study of over 100 infants for whom EEG was recorded when listening to nursery rhymes at 4, 7 and 11 months, Attaheri et al. (2022) found that a greater theta-delta power ratio at 11 months was associated with poorer vocabulary outcomes at 24 months. Interestingly, the large increase in spectral power in the delta band found in the current study accompanied by the smaller reduction in the theta band reduces the theta-delta power ratio for children with dyslexia. Accordingly, this change in neural dynamics could enhance language processing by children with dyslexia. However, as the planned intervention for the children in the current study was curtailed by the COVID-19 pandemic, it was not possible to check this possibility for the current participants.

As noted, *a priori* and following TS theory, EME speech was expected to change neural speech processing in the phase domain. The absence of changes in the phase domain may suggest that cortical tracking *per se* was not affected instantaneously by hearing EME speech in the current study. In contrast to the data for dyslexic adults reported by Lizarazu et al. (2021a), we did not find group differences in phase-locking values. However, it is likely that repeated experience with EME speech would be required to affect neural phase locking in children. Any instantaneous effects may be very small and given that MEG data with children is noisier than with adults, may be more difficult to detect. In the intervention study with pre-readers reported by Van Herck et al. (2022), a 12-week intervention with EE speech did improve ART discrimination in children at risk for dyslexia. However, the study did not measure any possible consequences of improved ART discrimination on cortical speech tracking. In the current study, the predicted right-lateralised effects of listening to the EME speech were not found. However, *post hoc* inspection of the marginal interaction effects (see figures) suggested that the lack of clear hemisphere effects was potentially due to a crossover effect for the children with dyslexia in the delta band response. For EME speech, spectral power was 0.40 in the right hemisphere for the children with dyslexia compared to 0.23 for the control children, and 0.39 in the left hemisphere for the children with dyslexia compared to 0.21 for the control children (see Figure 4). This change in delta power meant that the theta-delta power ratio was reduced in both hemispheres for children with dyslexia (see Table 2). Accordingly, we tentatively propose the theta-delta power ratio as a potential mechanism underpinning individual differences in phonological processing in dyslexia (Araújo et al., 2022). Future studies could consider exploring changes to this ratio as a potential target for remediation. Such studies could also investigate whether the higher spectral power in delta found here is instead an acoustic (low-level) effect caused by more pronounced acoustic information being present. A future study may also be able to disentangle the potentially compensatory hemisphere effects for children with dyslexia. It is entirely plausible that a study with higher statistical power may be able to disentangle hemispheric effects.

The study has several limitations. The obvious limitation is that the planned 20 sessions of intervention with EME speech could not be delivered to the participants due to the COVID-19 pandemic, and consequently that post-intervention neural imaging and assessments of phonological processing and reading could not be carried out. Accordingly, further research is required to ascertain whether the changed theta-delta ratio observed in response to EME speech in the current study improved children’s phonological skills and affected their reading performance and whether this change is temporary or exists over longer time scales. It is also possible that the EME algorithm could be further optimised. Although the EME speech did change neural speech processing for both children with dyslexia and control children, the effects were limited to the spectral domain, whereas TS theory is focussed on the phase alignment of the intrinsic oscillations and the incoming stimulus (Goswami, 2011). Nevertheless, neural phase and power dynamics are still poorly understood, and it is possible that the consistent delta-band speech encoding impairments found in children with dyslexia (Molinaro et al., 2016; Power et al., 2016; Di Liberto et al., 2018; Destoky et al., 2020, 2022; Mandke et al., 2022) in part reflect delta-theta power dynamics. It is also possible that repeated experience with EME speech is required before speech processing by the dyslexic brain is affected in the temporal domain. A study using EME speech and a 10-week rhythmic oral language intervention is currently in progress with a new group of participants and should throw some light on this possibility. Finally, it may be that enhancing the speech envelope is not the best way to remediate the phonological problems shown by many individuals with dyslexia.

In conclusion, neurophysiological studies of children with dyslexia have identified systematic impairments in envelope tracking in the delta band, which could potentially be remedied by enhancing selected aspects of the speech signal that is heard by affected children. This study provides one indication that EME speech changes neural speech processing mechanisms for children with dyslexia. Further studies are now required to investigate the potential of EME algorithms to improve the core phonological deficit that is one cognitive hallmark of developmental dyslexia across languages, as well as to disentangle temporal and spectral effects and potential lateralisation differences. A systematic comparison of the EME algorithm used here, the EE algorithm developed by Van Hirtum et al. (2019, 2021) as well as other possible EE algorithms, could throw further light on whether the cortical tracking deficits that appear to characterise children with dyslexia across languages can be ameliorated by enhancing selected features of the speech envelope.

## Data availability statement

This is an ongoing study and the data are still being analysed by the authors. As the data were obtained from children, our ethics approval does not give us explicit permission to make it publicly available.

## Ethics statement

The studies involving human participants were reviewed and approved by the Psychology Research Ethics Committee of the University of Cambridge, UK. Written informed consent to participate in this study was provided by the participants’ legal guardian/next of kin.

## Author contributions

KM: conceptualisation, methodology, investigation, software, data curation, formal analysis, visualisation, writing—original draft, and review and editing. SF: formal analysis, software, investigation, validation, visualisation, and writing—review and editing. AM, GF, FG, and AW: investigation and data curation. JG: conceptualisation, validation, and writing—review and editing. UG: conceptualisation, resources, methodology, investigation, project administration, supervision, funding acquisition, writing—original draft, and review and editing. All authors contributed to the article and approved the submitted version.

## Footnotes

1
https://www.neurobs.com/

2
http://neuroimage.usc.edu/brainstorm
