# Supplementary material for: Neural responses to natural and enhanced speech edges in children with and without dyslexia
Source: Front Hum Neurosci. 2023 Sep 28;17:1200950. doi: 10.3389/fnhum.2023.1200950 (PMC10571917; doi:10.3389/fnhum.2023.1200950)
Supplement: Supplementary file 1 [file Data_Sheet_1.pdf]

Neural responses to natural and enhanced speech edges in children with and without dyslexia

Kanad Mandke[*a], Sheila Flanagan[a], Annabel Macfarlane[a], Georgia Feltham[a], Fiona Gabrielczyk[a], Anji Wilson[a], Joachim Gross[b], Usha Goswami[a]

[a] *Centre for Neuroscience in Education, Department of Psychology, University of Cambridge, Cambridge, United Kingdom*
[b] *Institute for Biomagnetism and Biosignal Analysis, University of Münster, Münster, Germany*

[*] **Corresponding author**
Dr Kanad Mandke
Centre for Neuroscience in Education
Department of Psychology
University of Cambridge
Cambridge
CB2 3EB
United Kingdom
Email: knm27@cam.ac.uk

Supplementary material

Table S1 28 Channel ERBn bandpass filter bank edge frequencies. Note first filter is low pass and output not used.

Edge Number	Edge (Hz)
1 (low-pass)	100
2	137
3	179
4	225
5	277
6	334
7	398
8	470
9	549
10	638
11	736
12	846
13	969
14	1105
15	1257
16	1426
17	1614
18	1824
19	2057
20	2317
21	2607
22	2930
23	3289
24	3689
25	4135
26	4631
27	5184
28	5800
29	6486
30	7250

Figure S1. Bank of 28 overlapping ERB_n filters spanning 100 to 7250 Hz

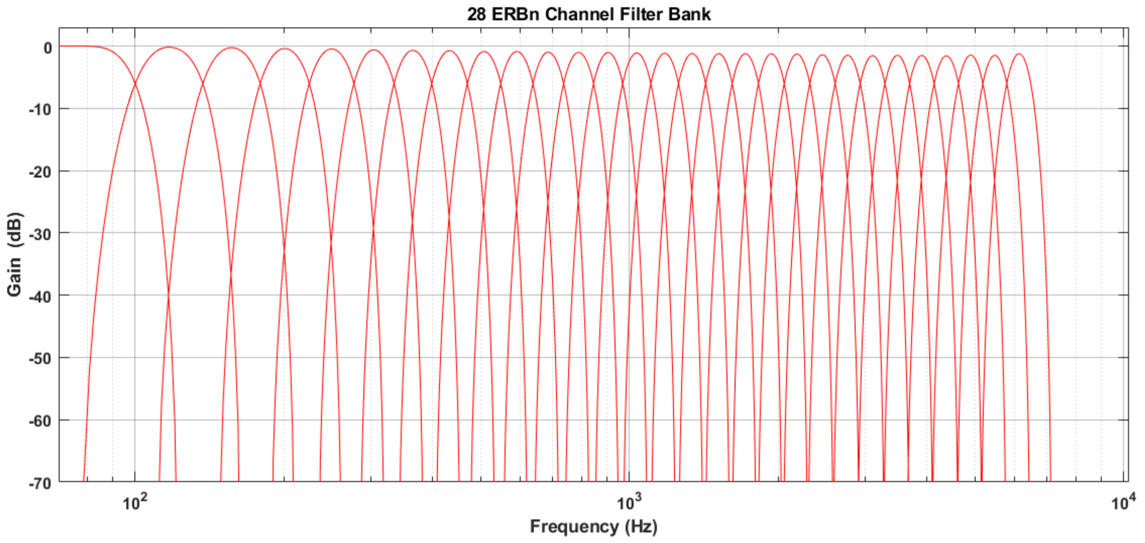

S2 Background measures: Standardised tests of reading, spelling, vocabulary, and IQ

Standardised tests were used in schools to assess cognitive development prior to the imaging session. Four subscales from the WISC-V (Weschler Intelligence Scales for Children – Fifth UK Edition) were administered. These included two verbal (vocabulary and similarities) and two non-verbal (block design and matrix reasoning) scales. Full-scale IQ was then estimated following the approach of (Aubry & Bourdin, 2018). In addition, the British Picture Vocabulary Scale (BPVS3) was administered to assess receptive vocabulary and the British Ability Scales (BAS) and the Test of Word Reading Efficiency (TOWRE-2) were administered to assess reading and spelling skills. A psychoacoustic measure of rise time sensitivity based on a synthetic syllable was also used.

1. *WISC Vocabulary, Similarities, Matrix Reasoning and Block Design Tests.*
   These four sub-tests of the WISC-V (Wechsler Intelligence Scales for Children) are used here to provide an estimate of full-scale intelligence. A brief description of these tests is provided below (Weschler, 2014).
   In the *Vocabulary* task, the child is asked to define a provided word. For picture items, the child names the depicted object. For verbal items, the child defines the word that is read aloud. There are 29 items with a maximum score of 54. A scaled score between 1 and 19 can then be computed, with the mean scaled score being 10.
   In the *Similarities* task, the child is given two words orally that name common objects or concepts and is asked to say how they are similar. There are 23 items, and the maximum raw score is 44. A scaled score between 1 and 19 can then be computed, with the mean scaled score being 10.
   In the *Matrix Reasoning* task, the children are shown an incomplete matrix or series of pictures and selects the option that completes the matrix or series. There are 32 items, and the maximum raw score is 32. A scaled score between 1 and 19 can then be computed, with the mean scaled score being 10.
   In the *Block Design* task, the child must recreate the design of a hand-made model or picture in a stimulus book within a specified time limit, using red and white blocks. There are 14 items with a total maximum score of 68. A scaled score between 1 and 19 can then be computed, with the mean scaled score being 10.
2. *British Picture Vocabulary Scales* (Dunn & Dunn, 2009). On a trial, the child is shown 4 pictures provided in a stimulus book, and the administrator says a word. The child must point to the picture that best illustrates the meaning of that word. The maximum raw score is 168 and a standard score can be computed with a mean of 100, S.D. 15.
3. *Test of Word Reading Efficiency* (TOWRE-2, Wagner et al., 2011). The TOWRE-2 consists of two subtests measuring rapid decoding of words (SWE, Sight Word Efficiency) and nonwords (PDE, Phonetic Decoding Efficiency). In each case, children are required to read aloud from a list of items graded in difficulty as many items as possible in 45 seconds, as quickly and as accurately as they can.
4. British Ability Scale – The British Ability Scales-3 (BAS-3, Elliot & Smith, 2011) is a set of standardized cognitive tasks that include measures of single-word reading and spelling. Children are presented with a sheet of single words that increase in difficulty to read aloud or are dictated a set of single words to spell that increase in difficulty, both with no time pressure. Assessment is halted after 6 consecutive errors, and standard scores can be computed with a mean of 100, S.D. 15.
5. The /ba/ rise discrimination task was presented as a child-friendly computer game based on an AXB “Dinosaur” threshold estimation program (following Dorothy Bishop, Oxford University). An adaptive staircase procedure (Levitt, 1971) using a

combined 2-down 1-up and 3-down- 1-up was used to converge rapidly on the child's auditory threshold. The sound tokens were resynthesized /ba/ speech syllables which were processed in Praat to create a continuum of 39 /ba/ rise stimuli. The /ba/ stimuli were based on those used in (Cumming et al., 2015) and adapted for this project by SF by the addition of random level roving of the standard stimuli in the range +/- 2.25 dB to remove loudness level as an alternative cue to the identification. The /ba/ syllable was 300 ms long with a flat f_0 (fundamental frequency) of 200 Hz. Three /ba/ sounds were played in sequence, with 3 cartoon "sheep", each jumping in turn. The second stimulus was the standard (X) and had a 10 ms amplitude onset rise time. The target stimulus was either A or B and had an onset rise time that ranged from the longest rise time at 150 ms to the shortest of 10 ms, in steps of 3.7 ms. The child was asked to find the sheep with the slowest or less distinct "Baa". A run consisted of 8 reversals or a maximum 40 trials. The threshold was estimated as the mean of the last 4 reversals.

References

Aubry, A., & Bourdin, B. (2018). Short forms of wechsler scales assessing the intellectually gifted children using simulation data. *Frontiers in Psychology*, *9*, 1–12. https://doi.org/10.3389/fpsyg.2018.00830

Cumming, R., Wilson, A., & Goswami, U. (2015). Basic auditory processing and sensitivity to prosodic structure in children with specific language impairments: a new look at a perceptual hypothesis. *Frontiers in Psychology*, *6*. https://doi.org/10.3389/fpsyg.2015.00972

Dunn, L. M., & Dunn, D. M. (2009). *The British picture vocabulary scale* (3rd edition). GL Assessment.

Elliot, C., & Smith, P. (2011). *British ability scale* (Third edition). GL Assessment.

Levitt, H. (1971). Transformed Up-Down Methods in Psychoacoustics. *The Journal of the Acoustical Society of America*, *49*(2B), 467–477. https://doi.org/10.1121/1.1912375

Wagner, R., Torgesen, J. K., & Rashotte, C. (2011). *Test of Word Reading Efficiency (TOWRE-2)* (Second edition).

Weschler, D. (2014). *Wechsler intelligence scale for children* (5th ed.). PsychCorp.
